# Artificial Urinary Sphincter: Long-Term Results and Patient Satisfaction

**DOI:** 10.1155/2012/835290

**Published:** 2012-03-26

**Authors:** Drogo K. Montague

**Affiliations:** Department of Urology, Center for Genitourinary Reconstruction, Glickman Urological and Kidney Institute, Cleveland Clinic, 9500 Euclid Avenue, Cleveland, OH 44195, USA

## Abstract

The published evidence concerning the safety, efficacy, and patient satisfaction for implantation of the current model of the artificial urinary sphincter (AS 800) in men with post prostatectomy urinary incontinence was the objective of this review. A Pub Med English language literature search from 1995 to 2011 was performed. A majority of men who undergo AUS implantation for post prostatectomy urinary incontinence achieve satisfactory results (0 to 1 pad per day). Infection rates range from 0.46 to 7%, cuff erosion rates range from 3.8 to 10%, and urethral atrophy ranges from 9.6 to 11.4%. Kaplan-Meier 5 year projections for freedom from any reoperation were 50% for a small series and 79.4% for a larger series. Kaplan-Meier projections for freedom from mechanical failure were 79% at 5 years and 72% at 10 years. In another series 10 year projections for freedom from mechanical failure were 64%. Although the artificial urinary sphincter (AUS) is the gold standard for the treatment of this disorder, most men will continue to need at least one pad per day for protection, and they are subject to a significant chance of future AUS revision or replacement.

## 1. Introduction

There is a wide range in the reported incidence of postradical prostatectomy urinary incontinence coming from individual series presumably due to inconsistent definitions of incontinence and differing modes of assessment. When large populations of postradical prostatectomy patients are surveyed, however, a more consistent pattern is observed. In a series of 1291 postprostatectomy patients, significant urinary incontinence persisted in 8.4% of men at eighteen months [1]. In a more recent NEJM study of 557 men at 12 months after radical prostatectomy, 24% were using pads, and 8% classified this as a moderate or big problem [2]. Urinary incontinence occurs less often after transurethral resection of the prostate being a significant problem in only 0.5% of 3885 men 2 months following surgery [3].

**Figure 1:**
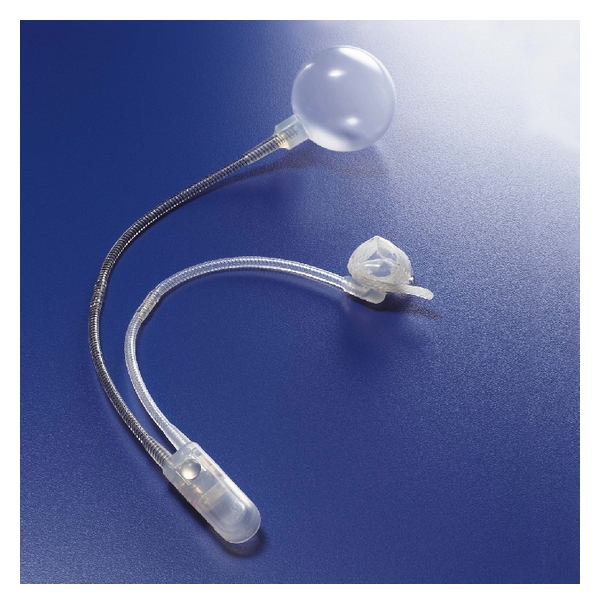
AS 800 (Used by permission of American Medical Systems, Minnetonka, MN, USA).

The artificial urinary sphincter (AUS) is widely regarded as the gold standard for the treatment of post prostatectomy urinary incontinence [4]. This prosthetic device was first introduced in 1973 [5], and during the next 10 years there were design changes resulting in 5 different models of the device [6]. The fifth model of the AUS, the AS 800 (American Medical Systems, Minnetonka, MN, USA) was introduced in 1983 and is still in use today. The AS 800 has 3 separate components: a cuff, a pressure regulating balloon, and a pump-control assembly (Figure 1). The components are implanted separately and connected by 2 tubing connectors. For post prostatectomy urinary incontinence, the cuff is placed around the bulbous urethra, the pressure regulating balloon is usually placed in the retropubic space, and the pump-control assembly is placed in the scrotum.

Since 1983 the basic design of the AUS has been unchanged; however, there have been numerous modifications to device components leading to both increased continence and longer device life. These component changes include narrow back cuffs for bulbous urethral use, smaller (3.5 and 4.0 cm) cuffs, surface-coated cuffs to reduce wear, kink-resistant tubing, tubing sleeves to reduce wear, and sutureless connectors to facilitate making connections and to reduce connector failures.

## 2. Materials and Methods

A PubMed English language literature search from 1995 through 2011 for keywords: artificial sphincter; urinary sphincter, artificial; prosthesis failure; prostatectomy/ae [adverse effects]; patient satisfaction; quality of life was performed. Thirteen articles were found where data relevant to patients with post prostatectomy incontinence could be separated from other AUS uses. These articles were examined to perform this paper.

## 3. Results

### 3.1. Efficacy

There is no standardization for reporting pre- and post-AUS levels of incontinence. In a study of 50 patients with a median followup of 23.4 months, the preoperative levels of incontinence were such that 70% wore an average of 6 diapers a day, and 24% wore an average of 7.4 pads per day [7]. After AUS implantation, 20% had complete continence. Of the remainder, 55% had leakage of a few drops, daily, and 22% had leakage of less than a teaspoonful.

In a study of 54 men with mean follow-up of 7.2 years, 54% were socially continent (0 to 1 pad per day). Mean pad score before AUS implantation was 2.75, and it decreased to 0.97 after AUS implantation [8].

In a group of 113 patients with mean follow-up of 73 months (range 20 to 170), 32% were pad free, 33% used 1 pad per day, 14% used 2 pads per day, 17% used 3 pads per day, and 5% used more than 3 pads per day [9].

In a study of 33 men aged 75 years or greater with an average follow-up of 5 years, mean pad use decreased from 6.7 pads per day (range 3–10) to 0.8 pads per day (range 0–2) [10].

In a study comparing 435 first time AUS implants to 119 repeat AUS implants, pad use of 0 to 1 day was noted in 90% of the primary implants and 82% of the secondary implants [11].

In 124 patients with a median follow-up of 6.8 years, 27.1% required no pads and 52.0% required 1 pad per day after AUS implantation [12].

In a cohort of 71 men with mean follow-up of 7.7 years (range 0.5–16), 27% used no pads, 32% used 1 pad, 15% used 1–3 pads, and 25% used more than 3 pads daily [13].

In a series of 40 men with mean follow-up of 53.4 ± 21.4 months (range 27–132), pad count decreased from 4.0 ± 0.9 to 0.62 ± 1.07 per day, and on a visual analog scale the impact of the incontinence decreased from 5.0 ± 0.7 to 1.4 ± 0.93 [14].

### 3.2. Cuff Erosion

Cuff erosion (Table 1) occurring within the first few weeks or months following AUS implantation is usually due to injury to the urethra when is mobilized prior to cuff placement. Late erosion usually occurs after a catheter has been inserted for a prolonged period without proper deactivation of the AUS. Unfortunately, very few reports of cuff erosion rates make the distinction between early and late cuff erosion.

### 3.3. Infection

AUS infection (Table 1) occurring without cuff erosion is not common. Most cuff erosions will lead to infection unless the erosion is detected early, and the AUS is removed before infection occurs. Most reporting of AUS infection fails to distinguish between infection alone and infection occurring as the result of cuff erosion.

### 3.4. Urethral Atrophy

When men achieve a satisfactory level of continence following activation of their AUS (0 to 1 pad per day) and later a gradual increase in pad use occurs, an AUS trouble shooting protocol should be employed [16]. If other causes of increasing incontinence have been ruled out by this protocol and the number of pump cycles to completely empty the cuff has increased, then urethral atrophy under the cuff has occurred. Possible revision procedures for this problem include cuff down sizing [17], tandem cuff placement [18], or transcorporeal cuff placement [19].

Urethral atrophy rates are shown in Table 1.

### 3.5. Mechanical Failure

For penile prosthesis implantation the American Urological Association Erectile Guidelines Committee has recommended that freedom from mechanical failure be reported in terms of Kaplan-Meier projections [20]. This allows meaningful comparisons within a single series and among several series where individual patient follow-up is variable. A similar recommendation should be adopted for reporting mechanical failure and other complications of AUS implantation. 

Kaplan-Meier reporting was used in one series where 66 AUS implantation patients were available for a mean follow-up of 41 months, The 5-year Kaplan-Meier projections for freedom from any reoperation was 50% and for freedom from any cuff revision was 60% [21]. In another series of 124 patients with median follow-up of 6.8 years, the 10 year Kaplan-Meier freedom from mechanical failure was 64% [12]. In a report of 530 men the 5 year Kaplan-Meier freedom from reoperation was 79.4%, for primary cases and 88% for revision surgeries [11]. In a fourth series of 39 men with bulbous urethral cuff AUS implantations, Kaplan-Meier freedom from mechanical failure at 5 years was 79% and at 10 years it was 72% [22].

Mechanical failure rates for the remaining studies are shown in Table 1.

### 3.6. AUS Implantation after Radiation Therapy

In one series, 58 men with AUS and no prior radiation (group 1) were compared to 28 with AUS after radiation therapy for prostate cancer (group 2) [23]. Mean follow-up was 31 ± 23 months for group 1 and 36 ± 21 months for group 2. Reoperation was required for 22.4% group 1 and 25% group 2. Urethral atrophy occurred in 14% in both groups. Urethral erosion occurred in 2% group 1 and 7% group 2. Infection occurred in 7% group 1 and none in group 2. None of these differences were statistically significant. The degree of continence (0-1 pad per day) was similar for both groups, 60% and 64%.

In another study, 76 men with AUS implantation and no radiation were compared to 22 men with AUS implantation after radiation [24]. Urethral atrophy, infection, and erosion were more common in the group with radiation (41%) compared to the group without radiation (11%).

### 3.7. Patient Satisfaction

Ideal satisfaction studies would be prospective and used standardized questionnaires administered pre- and post-operatively. In addition since incontinence may have a significant quality-of-life impact on the partner, including partners in these studies would be desirable. Unfortunately, nearly all studies are retrospective and use a variety of nonvalidated satisfaction scales.

In a study of 50 patients with a median follow-up of 23.4 months, 90% reported satisfaction, 96% would recommend AUS implantation to a friend, and 92% would have the AUS placed again [7].

In a study of 54 men with mean follow-up of 7.2 years, subjective improvement was 4.1, and overall satisfaction was 3.9 (scale 0 to 5) [8].

In a group of 113 patients with mean follow-up of 73 months (range 20 to 170), 28% were very satisfied, 45% were satisfied, 18% were neutral, 6% were dissatisfied, and 4% were very dissatisfied [9].

In 71 men with a mean follow-up of 7.7 years (range 0.5–16), 58% were very satisfied, 19% were satisfied, and 23% were unsatisfied [13]. 

## 4. Conclusions

Significant urinary incontinence following radical prostatectomy which persists for more than one year occurs in as many as 8% of men. Although the AUS is the gold standard for the treatment of this disorder, most men will continue to need at least one pad per day for protection, and they are subject to a significant chance of future AUS revision or replacement.

Current reporting of AUS implantation results is far from ideal. Freedom from mechanical failure and other complications should be reported in terms of Kaplan-Meier projections. Quality-of-life and patient satisfaction studies should be prospective, include partners, and use validated questionnaires.

## Figures and Tables

**Table 1:** 

Reference	*N*	Mean followup (months)	Infection (%)	Cuff erosion (%)	Urethral atrophy (%)	Device failure (%)	Removal/revision (%)
Lai et al. [15]	176	36.5	5.5	6.0	9.6	6.0	27.1
Kim et al. [12]	124	81.6	7	10		29	37
Raj et al. [11]	554	68	0.46	3.8	11.4	5.6	21.4
Gousse et al. [13]	71	92.4	1.4	4		25	29

## References

[1] Stanford JL, Feng Z, Hamilton AS (2000). Urinary and sexual function after radical prostatectomy for clinically localized prostate cancer: the prostate cancer outcomes study. *Journal of the American Medical Association*.

[2] Sanda MG, Dunn RL, Michalski J (2008). Quality of life and satisfaction with outcome among prostate-cancer survivors. *New England Journal of Medicine*.

[3] Mebust WK, Holtgrewe HL, Cockett ATK, Peters PC (1989). Transurethral prostatectomy: immediate and postoperative complications. A cooperative study of 13 participating institutions evaluating 3,885 patients. *Journal of Urology*.

[4] Herschorn S, Bruschini H, Comiter C (2010). Surgical treatment of stress incontinence in men. *Neurourology and Urodynamics*.

[5] Scott FB, Bradley WE, Timm GW (1973). Treatment of urinary incontinence by implantable prosthetic sphincter. *Urology*.

[6] Montague DK (1984). Evolution of implanted devices for urinary incontinence. *Cleveland Clinic Quarterly*.

[7] Litwiller SE, Kim KB, Fone PD, White RW, Stone AR (1996). Post-prostatectomy incontinence and the artificial urinary sphincter: a long-term study of patient satisfaction and criteria for success. *Journal of Urology*.

[8] Haab F, Trockman BA, Zimmern PE, Leach GE (1997). Quality of life and continence assessment of the artificial urinary sphincter in men with minimum 3.5 years of followup. *Journal of Urology*.

[9] Montague DK, Angermeier KW, Paolone DR (2001). Long-term continence and patient satisfaction after artificial sphincter implantation for urinary incontinence after prostatectomy. *Journal of Urology*.

[10] O’Connor RC, Nanigian DK, Patel BN, Guralnick ML, Ellision LM, Stone AR (2007). Artificial urinary sphincter placement in elderly men. *Urology*.

[11] Raj GV, Peterson AC, Toh KL, Webster GD (2005). Outcomes following revisions and secondary implantation of the artificial urinary sphincter. *Journal of Urology*.

[12] Kim SP, Sarmast Z, Daignault S, Faerber GJ, McGuire EJ, Latini JM (2008). Long-term durability and functional outcomes among patients with artificial urinary sphincters: a 10-year retrospective review from the University of Michigan. *Journal of Urology*.

[13] Gousse AE, Madjar S, Lambert MM, Fishman IJ (2001). Artificial urinary sphincter for post-radical prostatectomy urinary incontinence: long-term subjective results. *Journal of Urology*.

[14] Trigo Rocha F, Gomes CM, Mitre AI, Arap S, Srougi M (2008). A prospective study evaluating the efficacy of the artificial sphincter AMS 800 for the treatment of postradical prostatectomy urinary incontinence and the correlation between preoperative urodynamic and surgical outcomes. *Urology*.

[15] Lai HH, Hsu EI, Teh BS, Butler EB, Boone TB (2007). 13 years of experience with artificial urinary sphincter implantation at Baylor College of Medicine. *Journal of Urology*.

[16] Montague DK, Angermeier KW (2001). Artificial urinary sphincter troubleshooting. *Urology*.

[17] Saffarian A, Walsh K, Walsh IK, Stone AR (2003). Urethral atrophy after artificial urinary sphincter placement: is cuff downsizing effective?. *Journal of Urology*.

[18] Kowalczyk JJ, Spicer DL, Mulcahy JJ (1996). Long-term experience with the double-cuff AMS 800 artificial urinary sphincter. *Urology*.

[19] Guralnick ML, Miller E, Toh KL, Webster GD (2002). Transcorporal artificial urinary sphincter cuff placement in cases requiring revision for erosion and urethral atrophy. *Journal of Urology*.

[20] Montague DK, Jarow JP, Broderick GA (2005). Chapter 1: the management of erectile dysfunction: an AUA update. *Journal of Urology*.

[21] Clemens JQ, Schuster TG, Konnak JW, McGuire EJ, Faerber GJ (2001). Revision rate after artificial urinary sphincter implantation for incontinence after radical prostatectomy: actuarial analysis. *Journal of Urology*.

[22] Venn SN, Greenwell TJ, Mundy AR (2000). The long-term outcome of artificial urinary sphincters. *Journal of Urology*.

[23] Gomha MA, Boone TB (2002). Artificial urinary sphincter for post-prostatectomy incontinence in men who had prior radiotherapy: a risk and outcome analysis. *Journal of Urology*.

[24] Walsh IK, Williams SG, Mahendra V, Nambirajan T, Stone AR (2002). Artificial urinary sphincter implantation in the irradiated patient: safety, efficacy and satisfaction. *British Journal of Urology International*.

